# Supplementary material for: Structure supports function: Informing directed and dynamic functional connectivity with anatomical priors
Source: Netw Neurosci. 2022 Jun 1;6(2):401–19. doi: 10.1162/netn_a_00218 (PMC9205420; doi:10.1162/netn_a_00218)
Supplement: Supplementary file 3 [file netn-06-401-s003.pdf]

Pascucci, D., Rubega, M., Rué-Queralt, J., Tourbier, S., Hagmann, P. & Plomp, G. (2021). Supporting information for "Structure supports function: informing directed and dynamic functional connectivity with anatomical priors." *Network Neuroscience*. Advance publication. https://doi.org/10.1162/netn_a_00218

Selection of the maximum scaling range based on cross-validation across trials

In ordinary least-squares problems, a common approach to select a regularizing constant is the method of cross-validation. Classic cross-validation approaches are based on the methods of leave-one-trial out or train-test splitting. To accommodate the multi-trial definition of the STOK and si-STOK filters, which typically use information from all the trials to recover the 'average' dynamics (Milde et al., 2010), we used a simple train-test splitting approach.

The si-STOK was trained on half of the trials (selected as the even trials) and the predicted trial average was compared against the average of the remaining half of the trials (test set, selected as the odd number of trials). The predicted trials average was estimated by averaging single-trial predictions of the signals obtained at each time step of the si-STOK recursion. This procedure was reiterated for a set of maximum values of the scaling range (from 0.001 to 1, in 10 log steps). For each maximum of the scaling range, we then estimated the norm of the residuals (predicted minus test average) and evaluated the obtained curve (norm of residuals over maxima of the scaling range) for the selection of the maximum value to use for scaling SC matrices. A reasonable choice is a point near the 'knee' of the curve where residuals reach their minimum and remain stable thereafter. This corresponds to the minimum amount of regularization required to minimize the prediction error of unseen data, without excessive shrinkage of the filter's coefficients.

We applied this procedure to the human EEG data (face-only condition). As Figure S3 shows, the value of 0.1 chosen ad-hoc as the maximum scaling range across our tests falls well within the knee of the curve, indicating a point where prediction residuals are minimized without excessive regularization.

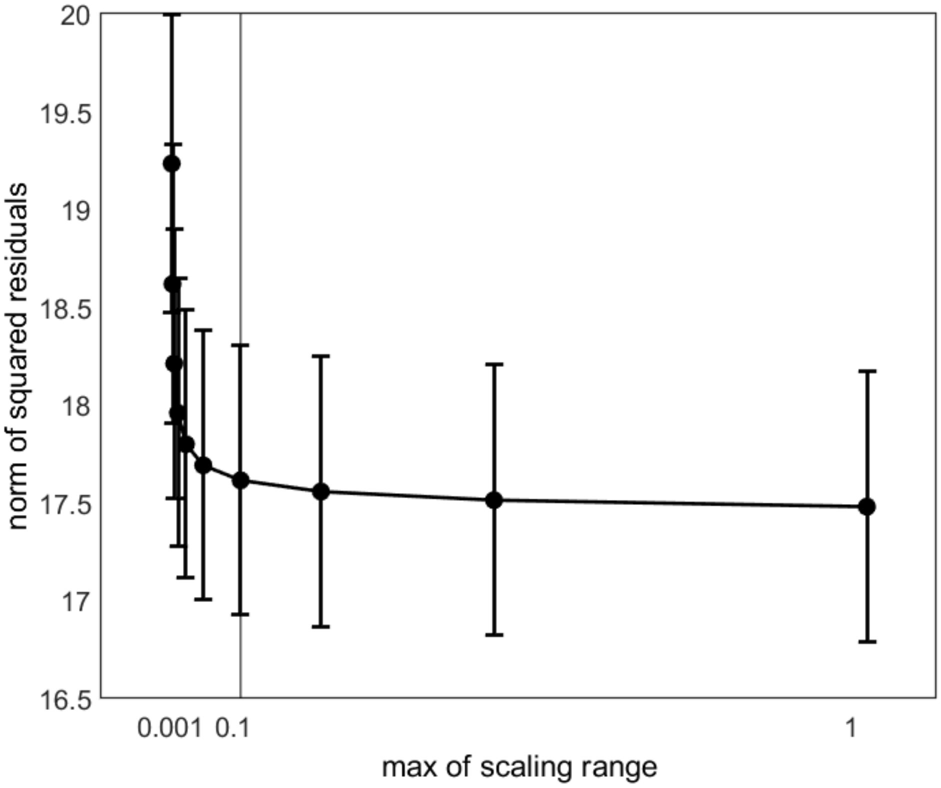

Figure S3. Maximum scaling range of SC in the si-STOK filter, evaluated as a function of the norm of squared residuals in a train-test splitting cross-validation routine. Data are from the human EEG dataset (19 subjects performing the face detection task, only trials for the condition with faces were used here). The vertical line indicates the value chosen ad-hoc and used throughout the manuscript. Error bars are 95% CI.
